# Supplementary material for: Content-rich biological network constructed by mining PubMed abstracts
Source: BMC Bioinformatics. 2004 Oct 8;5:147. doi: 10.1186/1471-2105-5-147 (PMC528731; doi:10.1186/1471-2105-5-147)
Supplement: Additional File 5 — The original Chilibot query results of the term "long-term potentiation (LTP)" and 22 other terms, limiting the latest references analyzed to the years 1990, 1995, 2000, and 2004. [file 1471-2105-5-147-S5.bz2 › chilibotAdditionalFile5/ltp2000/html/LTP_PI-3K.html]

 


 **LTP** and **PI-3K** 
  
Found 1 abstracts in PubMed,  **1 abstracts were retrieved and analyzed**.  


---

 Search Google  |
 PDF files only 
|  EDU domain only 

---

**Interactive relationship** (e.g. stimulation, inhibition, etc)

**Parallel relationship** (e.g. studied together, co-existance, homology, etc.)

- The evidence presented is consistent with the hypothesis that PI 3 kinase  [ **PI-3K** ]  activation, possibly by NGF, plays a role in expression of  **LTP**  in dentate gyrus.  Ref: 10728885 Neuropharmacology, 2000
- In this study, we have investigated the role of PI 3 kinase  [ **PI-3K** ]  in the expression of long term potentiation  [ **LTP** ]   **LTP**  in perforant path granule cell synapses of the rat.  Ref: 10728885 Neuropharmacology, 2000
